# The Psychological Science of Pandemics: Contributions to and Recommendations for Social, Educational, and Health Policy

**DOI:** 10.1177/17456916231216113

**Published:** 2024-02-20

**Authors:** Dolores Albarracin, Norbert Schwarz

**Affiliations:** 1 Annenberg School for Communication, University of Pennsylvania; 2 Department of Psychology, University of Southern California; 3 Marshall School of Business, University of Southern California

As we sail through the normalcy of the post-pandemic period, it is easy to forget that at this time in 2020, governments all over the world were scrambling to decide how to trace and control the spread of infections, what to communicate to the public, for how long to close schools, and what vaccination policies to implement, all while scientists were cooperating to produce vaccines and treatments against a backdrop of political polarization and intergroup conflict in many countries. In many cases, health, education, and related policies were enacted with little if any acknowledgment of the psychological principles that drive human behavior. The difficulties using psychological knowledge in these decisions stemmed in part from the emergency created by the pandemic. In addition, the body of knowledge relevant to policies during a pandemic was dispersed, and our scientists and scientific societies had not led efforts to generate and synthesize the data necessary for this and other emergencies.

*The Psychological Science of Pandemics: Contributions to and Recommendations for Social, Educational, and Health Policy* is a collection of eight articles that describe how psychological science can help to understand and address infectious disease spread while preserving well-being, learning, and constructive social interactions. The special issue includes articles that (a) analyze key policy questions, (b) succinctly review the relevant psychological literature, (c) critique actual policies implemented during the pandemic, and (d) propose research and policy questions that psychologists might address in the future. In addition, the articles either make or imply the recommendations we summarize in Table 1.

**Table 1.** Policy Recommendations

Area and recommendation no.	Recommendation	Special issue article
We are in this together		
1	A shared threat requires a shared response. Communication strategies that emphasize a valued overarching identity can facilitate this.	Van Bavel et al. (2023) Albarracin et al. (2024)
2	Buy-in from a broad range of political elites, opinion leaders, and influencers can reduce the adverse impact of political polarization and intergroup conflict.	Van Bavel et al. (2023) Butterworth et al. (2023) Stern and Ruisch (2023)
3	Nonpartisan experts should be the leaders and faces of the public health response.	Van Bavel et al. (2023)
4	Broad cooperation needs to be encouraged and facilitated. Amplify positive sum helping by making help easy to give, emphasizing the benefits of helping, and ensuring that actions are indeed helpful.	Butterworth et al. (2023)
Knowledge communication		
1	Communicate a mental model of community transmission.	Albarracin et al. (2024)
2	Communicate disease prevalence rather than changes in infections, particularly when changes are positive.	Albarracin et al. (2024)
3	Use metaphors and analogies to reach populations with different educational levels. But avoid “war” metaphors—they do not focus people on prevention measures.	Albarracin et al. (2024) Stern and Ruisch (2023)
4	In discussing the origins of a virus, avoid stereotypes and language that invites group blame.	Stern and Ruisch (2023)
Science communication		
1	Avoid communicating a false sense of consensus about complex issues such as the duration of immunity after a new vaccine is developed.	Albarracin et al. (2024)
2	Communicate consensus about personal health decisions by experts, including how many of them have been vaccinated against a disease.	Albarracin et al. (2024)
3	Provide a realistic image of science, emphasizing that science is characterized by responsiveness to new data rather than timeless truths.	Mertens et al. (2023) Albarracin et al. (2024)
4	Acknowledge uncertainty in public health communication.	Mertens et al. (2023)
Curbing misinformation		
1	When fringe positions are presented, avoid repeating them and highlight the correct consensus information through weight-of-evidence statements.	Albarracin et al. (2024)
2	Prebunk when possible.	Albarracin et al. (2024)
3	Debunk frequently disseminated claims.	Albarracin et al. (2024)
4	Ignore misleading claims that have received little attention.	Albarracin et al. (2024)
5	Consider methods in addition to debunking when an issue is politically polarized.	Albarracin et al. (2024)
6	Keep in mind that highlighting the presence of misinformation in the media can decrease trust in all media, which can hurt effective communication.	Albarracin et al. (2024)
7	To reduce the availability of misinformation, encourage scientists and the community to share accurate information for their mutual benefit.	Butterworth et al. (2023)
8	Create wikis for data sharing between scientists and the public to allow scientists to share discoveries and ask questions that encourage the community to provide accurate information.	Butterworth et al. (2023)
9	Rather than censoring misinformation or excluding their purveyors, connect mis/disinformation shared within the community with fact-based sources that people can access themselves.	Butterworth et al. (2023)
Communication for behavioral change		
1	Connect public health recommendations with valued social identities and culturally fluent behaviors.	Albarracin et al. (2024)
2	Aim for buy-in from political elites on all sides to avoid a politically polarized response to recommended behaviors.	Van Bavel et al. (2023)
3	Aim to reach across echo chambers; buy-in from political elites can facilitate this.	Van Bavel et al. (2023)
4	Target determinants of behavior change instead of merely focusing on correcting salient misinformation.	Albarracin et al. (2024)
5	Motivate adaptive behavior by providing information that increases a sense of controllability and predictability.	Mertens et al. (2023)
6	Communicate positive norms and positive changes in norms.	Albarracin et al. (2024)
Managing fear		
1	Address realistic sources of fear such as lack of health care access among ethnic minorities and other vulnerable groups.	Mertens et al. (2023)
2	Provide mental health support to counter the negative effects of fear among populations that continue to experience high levels of anxiety and isolate themselves despite the availability of effective vaccines and treatments.	Mertens et al. (2023)
Public health policies for behavioral change		
1	Communicate about positive policies such as increased funding for vaccination.	Albarracin et al. (2024)
2	Use mandates.	Albarracin et al. (2024) Fayaz-Farkhad and Jung (2023)
3	Consider using case managers to support or accompany vulnerable members of the population as they navigate the process from making appointments to getting to the vaccine.	Albarracin et al. (2024)
4	Expand free health care to cover any vaccine side effects and coverage for postvaccination recovery days.	Albarracin et al. (2024)
5	Introduce vaccination passports.	Fayaz-Farkhad and Jung (2023)
Contact tracing		
1	Educate the population about digital contact tracing and how disease spreads ahead of time.	Garry et al. (2023)
2	Make health care systems more digitally aware and better integrated with digital contact tracing.	Garry et al. (2023)
3	Integrate digital contact tracing into personal devices already associated with health (e.g., Garmin and Apple watches).	Garry et al. (2023)
4	Improve the user experience of digital contact-tracing apps and do so in ways that encourage trust in them.	Garry et al. (2023)
5	Integrate contact-tracing apps with preventive messages.	Garry et al. (2023)
Enhanced cooperation		
1	Develop official data dashboards and virtual research teams early to allow diverse fields to collaborate and contribute to public health decisions.	Butterworth et al. (2023)
2	Create mutual-aid groups and mutual-aid apps that connect members of the community in reciprocally beneficial, timely, and local exchanges.	Butterworth et al. (2023)
3	Empower grassroots efforts and local authorities to make policies that benefit individuals in local, concrete situations.	Butterworth et al. (2023)
Schools		
1	Ensure that schools are equipped with technology that allows for easy transitions from in-person to virtual learning.	Mazrekaj and de Witte (2023)
2	Provide financial assistance as well as computers, after-school programs, and online tutoring to vulnerable families.	Mazrekaj and de Witte (2023)
3	Increase the scale of mental health and resilience programs for families with children.	Mazrekaj and de Witte (2023)
4	Foster social and emotional learning in schools.	Mazrekaj and de Witte (2023)
5	To reduce the effects of negative expectations, avoid stigmatizing labels such as “The COVID Generation” or “Generation C” or “The Lost Generation.”	Mazrekaj and de Witte (2023)

In the first article, “Health Communication and Behavioral Change During the COVID-19 Pandemic,” along with Daphna Oyserman, we consider critical issues related to public health communication and interventions to change behavior during the pandemic. We analyze the challenges and opportunities of communicating information about risk and necessary precautions, appealing to individuals from different cultural backgrounds, addressing misinformation, and changing behavior. The article provides recommendations about knowledge transmission, science communication, curbing misinformation, and changing behavior.

In “The Costs of Polarizing a Pandemic: Antecedents, Consequences, and Lessons,” van Bavel et al. review how political polarization shaped the response to, and the outcomes of, the COVID-19 pandemic in the United States. The authors detail how political polarization affected every aspect of the pandemic, from initial risk perceptions, spatial distancing, mask-wearing, and vaccination uptake to a mortality rate that differed dramatically between Democratic- and Republican-leaning counties. They conclude that the exceptionally high death toll in the United States was due, in large part, to political polarization, which shaped citizens’ protective behaviors. Their discussion raises questions for further research and offers policy recommendations, as summarized in Table 1.

In “Cooperation in the Time of Covid,” Butterworth et al. address altruism during a pandemic. Because human cooperation evolved in small groups, life in large, impersonal societies can produce a breakdown in altruistic behavior. This is particularly clear when people are not identifiable, when interactions are one-off, when self-interest is not tied to the interests of others, and when people are concerned about others not reciprocating. From this perspective, policies for managing pandemics should highlight superordinate goals, connect people and institutions over multiple interactions, and provide reputational markers for people who cooperate. The authors provide critical recommendations about misinformation and cooperation, which are summarized in Table 1.

In “Managing Fear During Pandemics: Risks and Opportunities,” Mertens et al. discuss the central roles of fear in adaptive risk judgments as well as its exacerbation in mental health problems such as anxiety disorders. On one end, a lack of fear can lead people to ignore government measures and take unnecessary risks, ultimately leading to infectious disease. On the other end, an excess of fear can trigger harmful effects such as unwarranted social anxiety, extreme isolation, and xenophobia. Implicit in Merterns et al.’s analysis are recommendations about misinformation, fear communication, and mental health services (see Table 1).

In “Do COVID-19 Vaccination Policies Backfire? The Effects of Mandates, Vaccination Passports, and Financial Incentives on COVID-19 Vaccination,” Fayaz-Farkhad and Jung conduct a systematic review of the evidence of three critical policies. Even though the policies are not without limitations, mandates, vaccination passports, and financial incentives did not backfire, as commonly feared. Instead, mandates and vaccine passports had large positive effects, whereas financial incentives had more variable, and typically shorter-lived, effects. Their rigorous analysis suggests the policy recommendations summarized in Table 1.

In “Hits and Misses: Digital Contact Tracing in a Pandemic,” Garry et al. cover traditional and digital contact tracing. The article discusses the memory challenges of traditional contact tracing, the promises of digital contact tracing, and the failures to deliver on this potential. They trace the failure to a lack of adequate interfaces with healthcare systems and the difficulties of individuals in both using and trusting contact-tracing apps. Several recommendations presented in Table 1 follow from the excellent lessons Garry and colleagues derive.

In “The Impact of School Closures of Learning and Mental Health of Children: Lessons From the COVID-19 Pandemic,” Mazrekaj and de Witte review how school closures of unprecedented scale and duration created both learning and mental-health deficits among children. They recommend greater attention to the intervention needs of marginalized children, personality tailoring in learning, and mental health interventions to reduce the deficits created during the pandemic, and avoidance of generational labels that stigmatize children. The important recommendations that follow from their analysis appear in Table 1.

In “How Do Pandemic Policies and Communication Shape Intergroup Outcomes? Initial Findings From the COVID-19 Pandemic and Open Questions for Research and Policy,” Stern and Ruisch discuss how government policies intended to curb pandemics, and the communication strategies used to implement them, can have an adverse impact on marginalized groups. Among other adverse effects, the pandemic was associated with increased anti-Asian bias and hate crimes against Asian individuals, more negative attitudes toward immigrants in general, and stronger endorsements of traditional gender roles. Stern and Ruisch offer informed conjectures about the processes involved, propose directions for future research, and note possible implications for future policy and communication strategies, as summarized in Table 1.

In combination, the contributions to this special issue illustrate the breadth of what psychological science has to offer to public health policy as well as the limits of the available knowledge. We are hopeful that the wide range of novel psychological research stimulated by the COVID-19 pandemic will advance our future understanding of human behavior in the context of severe health threats and contribute to better preparation for future pandemics.

## Transparency

*Action Editor:* Lora Park

*Editor:* Interim Editorial Panel

**ORCID iD:** Norbert Schwarz  https://orcid.org/0000-0002-8868-7067
